# Comparison of the Complete *Eragrostis pilosa* Chloroplast Genome with Its Relatives in Eragrostideae (Chloridoideae; Poaceae)

**DOI:** 10.3390/plants8110485

**Published:** 2019-11-09

**Authors:** Yamuna Somaratne, De-Long Guan, Nibras Najm Abbood, Liang Zhao, Wen-Qiang Wang, Sheng-Quan Xu

**Affiliations:** 1 College of Life Sciences, Shaanxi Normal University, Xi’an 710062, China; yamuna@snnu.edu.cn (Y.S.); guandelong@snnu.edu.cn (D.-L.G.); nibras.abbood@gmail.com (N.N.A.); 2 College of Life Sciences, Northwest A & F University, Yangling 712100, China; biology_zhaoliang@126.com; 3 College of Life Sciences, Yan'an University, Yan'an 716000, China

**Keywords:** *Eragrostis pilosa*, Eragrostideae, Chloridoideae, Poaceae, chloroplast genome

## Abstract

*Eragrostis* of the tribe Eragrostideae is a taxonomically complex genus, because of its polyploid nature and the presence of similar morphological characters among its species. However, the relationship between these morphologically indistinguishable species at the genomic level has not yet been investigated. Here, we report the complete chloroplast genome of *E. pilosa* and compare its genome structures, gene contents, simple sequence repeats (SSRs), sequence divergence, codon usage bias, and Kimura 2-parameter (K2P) interspecific genetic distances with those of other Eragrostideae species. The *E. pilosa* chloroplast genome was 134,815 bp in length and contained 132 genes and four regions, including a large single-copy region (80,100 bp), a small single-copy region (12,661 bp), and a pair of inverted repeats (21,027 bp). The average nucleotide diversity between *E. pilosa* and *E. tef* was estimated to be 0.011, and 0.01689 among all species. The minimum and maximum K2P interspecific genetic distance values were identified in *psaA* (0.007) and *matK* (0.029), respectively. Of 45 SSRs, eight were shared with *E. tef*, all of which were in the LSC region. Phylogenetic analysis resolved the monophyly of the sampled *Eragrostis* species and confirmed the close relationship between *E. pilosa* and *E. tef*. This study provides useful chlorophyll genomic information for further species identification and phylogenetic reconstruction of *Eragrostis* species.

## 1. Introduction

*Eragrostis* is a large genus comprising more than 350 species in the tribe Eragrostideae (subfamily Chloridoideae; family Poaceae), which includes three subtribes (Cotteinae (4 genera), Eragrostidinae (11 genera), and Uniolinae (5 genera) [1,2]. *Eragrostis* species are distributed in weedy disturbed areas and dry habitats of the world’s tropical and subtropical regions [3]. It is commonly called love grass or cane grass, and has significant uses in different areas. Most species belonging to this genus are used as animal feed. However, *E. tef* is a staple food in some countries, such as Ethiopia. *E. amabilis* is grown as an ornamental plant, and *E. cynosuroides* has religious meaning and is used in Hindu temples. Growing *E. bahiensis* can remove highly toxic radioactive atoms from the environment, and *E. curvula* is widely planted to prevent soil erosion.

Previously, all Eragrostideae species were thought to use the C4 photosynthetic pathway. Leaf blade anatomies common to the C4 pathway, including NAD-ME, PCK-like, and intermediate forms, are observed in the genus *Eragrostis*. Similar to other Chloridoideae, chloridoid-, panicoid-, *Pappophorum*-type, and intermediate bicellular microhairs are observed in *Eragrostis* species [4,5]. However, Ellis (1984) [6] reported non-C4 leaf anatomy in *E. walteri*. Moreover, carbon isotope analyses confirmed that *E. walteri* is a C3 plant, while phylogenetic analyses positioned it outside Chloridoideae in Arundinoideae, a subfamily comprised entirely of C3 species [7,8].

Wolf (1776) [9] described the first *Eragrostis* species, *E. minor*. However, despite its identification long ago, which species actually belong in this genus remains debatable. Due to its large size and wide geographic distribution, there has been no comprehensive taxonomic analysis of *Eragrostis*. Ingram et al. (2007) [3] suggested that *Eragrostis* is monophyletic, with the inclusion of Acamptoclados, Diandrochloa, and Neeragrostis. In 2003, Ingram and Doyle described the close relationship between *E. tef* and *E. pilosa* using the nuclear gene *waxy* and the plastid locus *rps16* [10]. 

*E. pilosa* is a common weed, native to Eurasia and Africa and distributed throughout tropical and subtropical areas of the world. The morphology of *E. pilosa* is very similar to *E. tef*, making them indistinguishable [11]. However, *E. pilosa* has been adapted to many climates, while *E. tef* has not. *E. pilosa* is an allotetraploid of hybrid origin that contains genes from other species. Ayele et al. (1999) [11] showed that *E. pilosa* and *E. tef* share many identical amplified fragment-length polymorphism markers. Therefore, they suggested that *E. tef* is a domesticate of *E. pilosa*, with few agronomic features altered during the domestication process. 

The plastid, which is maternally inherited and functions in photosynthesis, carbon fixation, and stress response, provides additional information for species identification and evolutionary study [12]. With the development of advanced technologies, the amplification and sequencing of plastid loci has been possible for many years. The availability of complete chloroplast (cp) genome sequencing data has expanded rapidly with the onset of next-generation sequencing (NGS) technology. To date, eight complete cp genome sequences belonging to subtribe Eragrostideae (*Eragrostis* (five species), *Enneapogon* (two species), and *Uniola* (one species)) are available in the National Center for Biotechnology Information (NCBI) database. However, comparison of these cp genomes has not been previously reported. Here, we present the complete cp genome of *E. pilosa*, and compare its structural variations, gene arrangements, and simple sequence repeat (SSR) distributions with the complete cp genomes of *E. tef* and seven other species from tribe Eragrostideae. Additionally, we introduce several SSR primers for future screening of *E. pilosa*. Our results demonstrate the diversity of species in tribe Eragrostideae. These data are consistent with previous studies suggesting close relationships between *E. pilosa* and other *Eragrostis* species. 

## 2. Results

### 2.1. Characteristic cp Genome Features of E. pilosa among Eragrostideae Species

**Figure 1:**
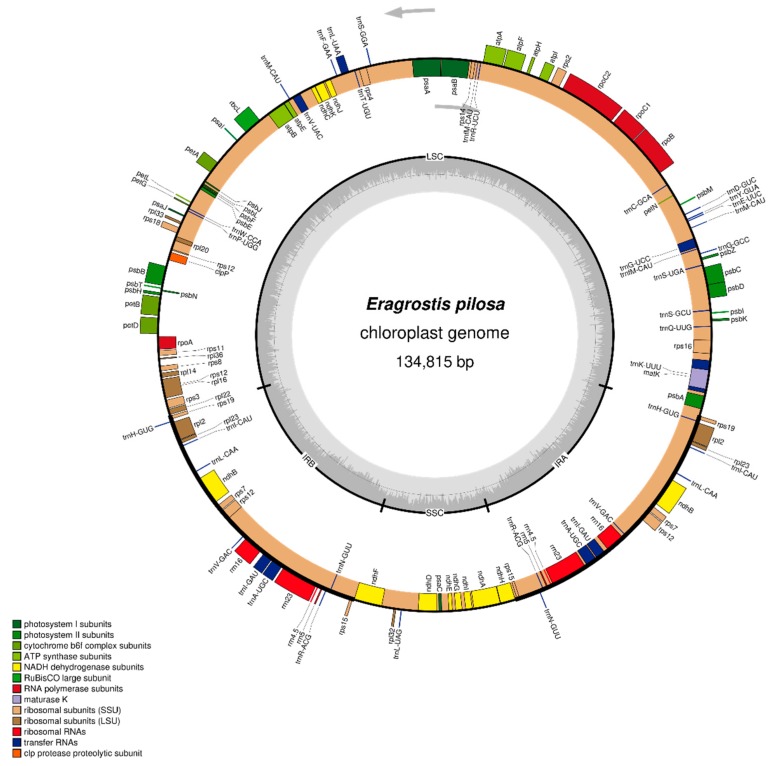
Gene map of *E. pilosa* chloroplast genomes. Genes on the inside of the map are transcribed in the clockwise direction and genes on the outside of the map are transcribed in the counterclockwise direction. The darker gray in the inner circle represents to GC content whereas the light gray corresponds to AT content. Different functional group of genes are shown in different colors.

The complete cp genome of *E. pilosa* was 134,815 bp long, and displayed a typical quadripartite structure containing a large single-copy (LSC) region (80,100 bp), a short single-copy (SSC) region (12,661 bp) and a pair of inverted repeat (IR) regions (21,027 bp). It consisted of 85 protein-coding genes, 39 tRNA genes, and eight rRNA genes, for a total of 132. Seven protein-coding genes (*rps15*, *ycf68*, *rps7*, *ndhB*, *rpl23*, *rpl2*, and *rps19*), eight tRNA genes (*trnN-GUU*, *trnR-ACG*, *trnA-UGC*, *trnI-GAU*, *trnV-GAC*, *trnL-CAA*, *trnI-CAU*, and *trnH-GUG*), and four rRNA genes were duplicated in the IR regions. In addition, *trnM-CAU* and *trnfM-CAU* were repeated in the LSC region. Ten protein-coding genes, including two duplicated genes (*rpl2* and *ndhB*) and eight tRNA genes, including two duplicated genes (*trnI-GAU* and *trnA-UGC*) contained one intron, whereas the protein-coding gene *ycf3* had two introns. Similar to most of the other angiosperms, the 5' end of *rps12* was located in the LSC region and 3' end was duplicated in the IR regions (Figure 1). 

The *E. pilosa* plastome sequence generated in this study was compared to all available complete plastomes from the tribe Eragrostideae. Six plastomes belonged to *Eragrostis*, two to *Enneapogon*, and one to *Uniola*. Of the Eragrostideae species, *E. tenellula* had the smallest cp genome (130,773 bp) while *E. walteri* had the largest (137,056 bp). The total number of genes varied among species, but *E. pilosa* and *E. tef* encoded an identical number of total genes, including the same protein-coding, tRNA, and rRNA genes. *E. tenellula* encoded the lowest total number of genes (124) due to loss of one copy of *trnfM-CAU* and the protein-coding genes *rps19, ycf68, rps15*, and *ndhH*. A copy of *trnfM-CAU* also lost in *En. caerulescens*, *E. setifolia*, and *E. walteri*. The additional *trnT-GGU* gene clustered with *trnM-CAU* in *U. paniculata*, *En. oblongus*, and *E. minor*. The protein-coding genes *rps19* and *ycf68* were also lost in *E. setifolia* and *En. caerulescens*, and loss of *ycf68* was also observed in *U. paniculata* and *E. walteri* (Table 1 and Table S1).

**Figure 2:**
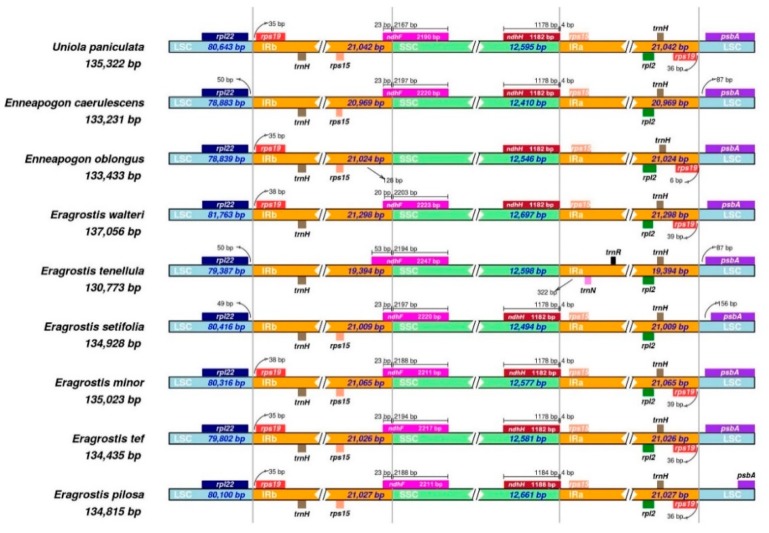
Comparison of LSC, SSC and IR borders among nine chloroplast genome of Eragrostideae.

The IR/SSC boundary regions of Eragrostideae species displayed some significant differences, even among species in the genus *Eragrostis*. *E. pilosa*, *E. tef*, and *E. minor* had similar IR/SSC boundary patterns, except for length variations in *ndhF* and *ndhH*. In *E. pilosa* and *E. minor*, *ndhF* was 2211 bp, but was 2217 bp in *E. tef*. This gene crossed the IRB/SSC boundary region by 23 bp in all three species. Furthermore, *ndhH* crossed the IRA/SSC region by 4 bp, and was 1188 bp long in *E. pilosa* and 1182 bp long in *E. tef* and *E. minor*. The *trnH* gene was located in the IRA region and duplicated in the IRB region, and was 75 bp in all species. The 282-bp *rps19* gene was present in the IRA region and repeated in IRB in all species except *E. setifolia*, *E. tenellula*, and *En. caerulescens* (Figure 2).

### 2.2. Codon Usage

The GC contents of the entire cp genomes, the coding sequences (CDS), and each codon position were similar in all species. The GC content in the plastomes and CDS were lower (approximately 38.3% and 39%, respectively) suggesting an abundance of AT regions. The mean GC content at the first codon position (GC1, 48.0%) was higher than the mean values at the second and third positions (GC2 and GC3; 39.1% and 30.0% respectively) in all species (Table 2). The average number of codons ranged between 14,407 (in *En. caerulescens*) and 14,431 (in *E. walteri*). In all species, leucine and cysteine were the most and least abundant amino acids, respectively. Methionine and tryptophan were each represented by only one codon, while all other amino acids were encoded by more than one codon in all Eragrostideae species. In *E. pilosa*, leucine was encoded by six synonymous codons (UUA (522), UUG (281), CUU (328), CUC (95), CUA (225), and CUG (77)), with relative synonymous codon usage (RSCU) values of 2.05, 1.1, 1.29, 0.37, 0.88, and 0.3, respectively (Table S2). 

### 2.3. Simple Sequence Repeats

SSRs in cp genomes are widely used in species identification, plant population genetics, and evolutionary studies [13,14]. In *E. pilosa*, 45 SSRs > 10 bp in length were detected, all of which were A/T and C/G mononucleotide repeats. The highest number of SSRs was detected in *E. walteri* (52), including 51 mononucleotide repeats and one dinucleotide repeat. The lowest number (41) was identified in *E. minor*. In all species, the SSRs were both mononucleotide or dinucleotide repeats, most were located in the LSC region, and A/T repeats were the most abundant type. All *Eragrostis* species except *E. walteri* contained SSRs incorporating C/G nucleotides. Compound SSRs also contained remarkably high A/T content, and in *E. pilosa*, only one compound SSR contained G nucleotides. *E. tef* and *E. walteri* had the highest number of compound SSRs containing A or T. The length of compound SSRs ranged from 22 to 200 bp in all species (Table 3). 

**Figure 3:**
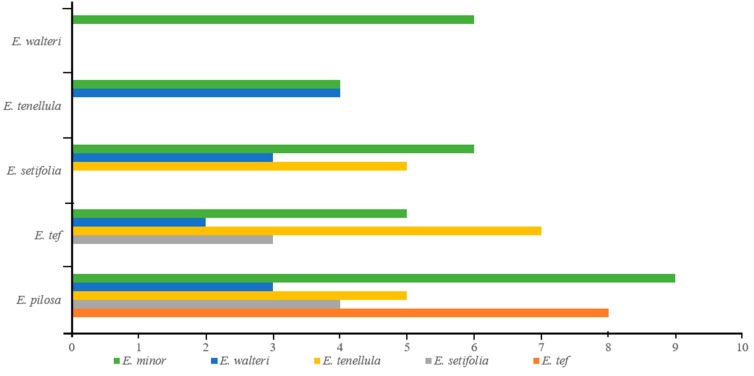
Shared SSRs among *Eragrostis* species.

Shared SSRs among *Eragrostis* species were calculated by aligning homologous regions in each pair of cp genomes. *E. pilosa* and *E. minor* shared the most SSRs (9), while *E. tef* and *E. walteri* shared the least (2). *E. pilosa* shared eight, four, and five SSRs with *E. tef*, *E. setifolia*, and *E. tenellula*, respectively, while *E. tef* and *E. minor* shared five (Figure 3 and Table S3). Ten *E. pilosa* SSRs were randomly selected, and primers were designed that can be used in future studies of the genetic diversity and population structure of these species (Table S4).

### 2.4. Sequence Divergence and Kimura 2-Parameter (K2P) Genetic Distance 

**Figure 4:**
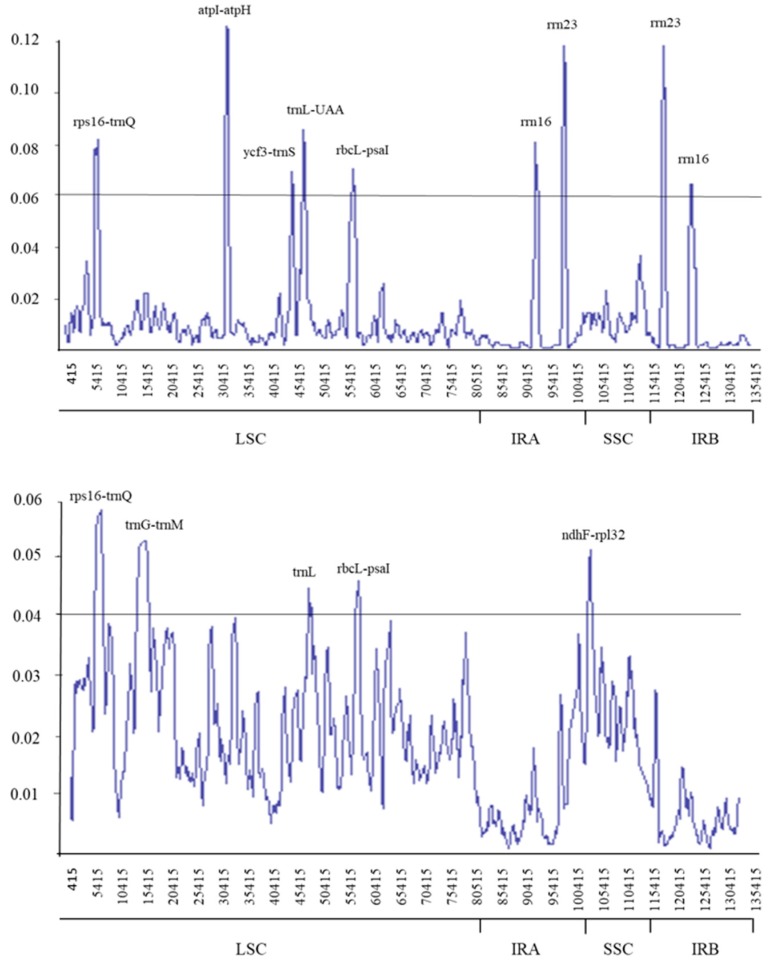
Sliding window analysis of the whole chloroplast genomes. (**top**) Pi among *E. pilosa* and *E. tef*. (**bottom**) Pi among *E. pilosa* and other eight species. Window length: 800 bp; step size: 200 bp. X-axis: position of the midpoint of a window. Y-axis: nucleotide diversity of each window.

**Figure 5:**
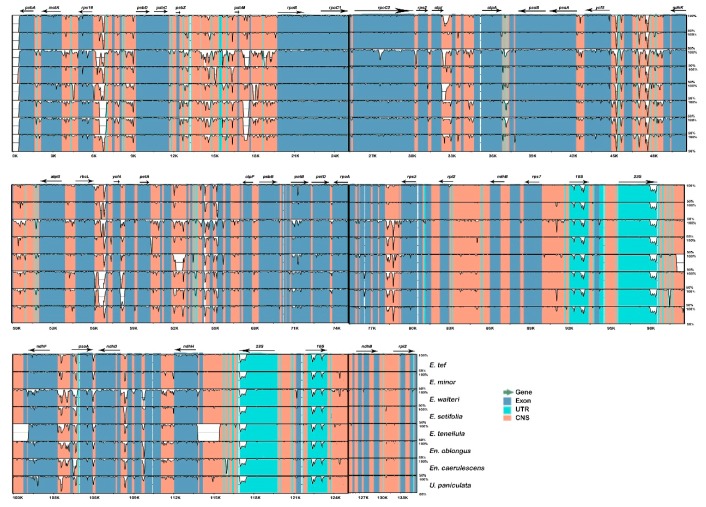
Sequence alignment plot comparing four cp genomes with *E. pilosa* as a reference. Genome regions are color coded as protein coding, rRNA coding, tRNA coding or conserved noncoding sequences. The vertical scale indicates the percentage identity, ranging from 50% to 100%.

Coding and non-coding regions that are highly variable between cp genomes are useful as potential DNA barcodes for species identification and phylogenetic reconstruction [15]. According to DNA Sequence Polymorphism (DnaSP), the estimated average nucleotide diversity (pi) value between *E. pilosa* and *E. tef* was 0.011. The highest variation was found in the LSC region (*atpI*-*atpH*) with a value of 0.125. Five regions in the LSC (*rps16*-*trnQ*, *atpI*-*atpH*, *ycf3*-*trnS*, *trnL*-*UAA*, and *rbcL*-*psaI*) showed high variability, with pi values > 0.06. The highest nucleotide diversity in the IR regions was in *rrn23* (pi=0.117) while *rrn16*, which clusters with *rrn23,* had a pi value > 0.063. For the nine cp genomes, pi values ranged from 0-0.05872, with an average of 0.01689. The loci *rps16*-*trnQ*, *trnG-trnM-trnT*, *trnL*, *rbcL-psaI*, and *ndhF-rpl32* were highly divergent among Eragrostideae species (pi > 0.04; Figure 4). The highest nucleotide diversity was observed in *rps16-trnQ* (0.05872). Furthermore, we performed a multiple sequence alignment between the nine complete cp genomes in mVISTA, using *E. pilosa* as a reference. (Figure 5). Similar to the estimated nucleotide diversity, the most divergent regions were located in the intergenic spacer regions of the LSC.

**Figure 6:**
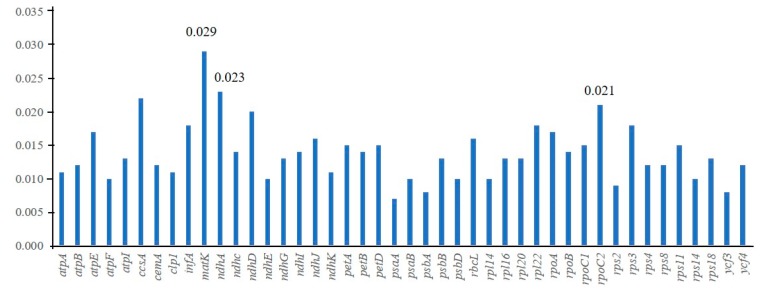
The K2P values for individual protein coding genes.

Using the sequences of 44 protein-coding genes, we calculated the interspecific K2P genetic distances among the nine species. Minimum and maximum K2P values were observed in *psaA* (0.007) and *matK* (0.029), respectively. The average K2P interspecific genetic distance was estimated at 0.014 (Figure 6). Pairwise comparisons of genetic divergence were estimated using the same protein-coding sequences. The lowest genetic divergence value was between *E. pilosa* and *E. minor* (0.003), while the highest divergence values (0.03) were between *E. walteri* and *En. caerulescens*/*En. oblongus*. Genetic divergence values between *E. pilosa* and *E. minor*, *E. tef*, *E*. *walteri, E. setifolia*, and *E. tenellula* were 0.003, 0.006, 0.028, 0.007, and 0.008, respectively, with the highest divergence observed between *E. pilosa* and *E. walteri*. When *Enneapogon* species were included, genetic divergence values with *Eragrostis* species were always high (ranging from 0.014 to 0.030). *U. paniculata* also showed relatively high genetic divergence with *Eragrostis* (ranging from 0.008 to 0.026) and *Enneapogon* (0.013) species. The genetic distance between *En. caerulescens* and *En. oblongus* was 0.002 (Table S5).

### 2.5. Phylogeny

**Figure 7:**
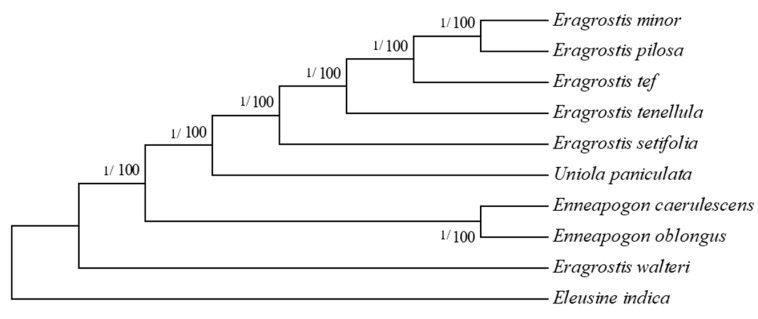
Phylogenetic trees based on ML and BI analysis on whole cp genome sequences Bootstrap values and posterior values are shown in each node.

**Figure 8:**
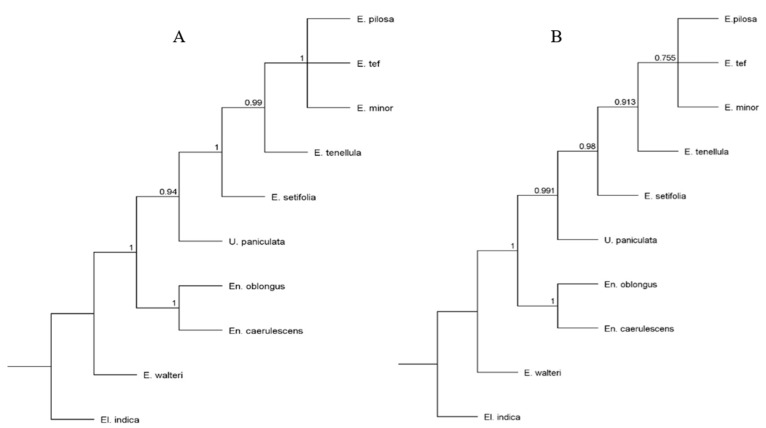
Phylogenetic trees based on divergence region and protein coding genes. (**A**) BI tree based on *rps16-trnQ* + *trnL*+ *rbcL-psaI* variable region; (**B**) BI analysis based on 30 protein coding genes. Posterior values are shown in each node.

The nine Eragrostideae species and the outgroup species *Eleusine indica* (NC_030486) from Cynodonteae were used for phylogenetic analysis. Similar topologies were observed in maximum likelihood (ML) and Bayesian inference (BI) generated trees based on complete cp genome sequences, with high bootstrap values for almost all relationships. *E. pilosa* was in the same clade as *E. minor*, and both were sisters of *E. tef* (Figure 7). BI trees based on highly divergent regions (*rps16-trnQ* + *trnL*+ *rbcL-psaI*) and 30 protein-coding genes of length > 600 bp were identical with high posterior probability values, and *E. pilosa*, *E minor*, and *E. tef* clustered together (Figure 8). All *Eragrostis* species except *E. walteri* had close relationships in all analysis. *E. walteri* was in the basal position followed by the *Enneapogon* species.

## 3. Discussion 

Chloroplasts are essential plant cell organelles that convert solar energy to usable chemical energy [12]. They are involved in the synthesis of most leaf proteins, fatty acids, amino acids, and secondary metabolites. The chloroplast enzyme ribulose-1,5-bisphosphate carboxylase (RbcL) is the most abundant protein on earth, and it is widely used in evolutionary studies of plants [16]. During evolution, specific cp genes have been lost in various species, genera, and families [17]. Therefore, comparing the cp genomes of plant species is useful to understand their evolutionary processes over time. The development of advanced technologies for cp genome sequencing has allowed many research groups to produce complete cp DNA sequences. In addition, no single DNA barcode exists that can distinguish all angiosperms from each other. Therefore, many recent studies have been conducted to detect appropriate barcodes to identify species, genera, and families using complete cp sequences [18].

All cp genomes used in this study had a typical quadripartite structure containing two copies of the IR region separating the SSC and LSC regions, similar to most other angiosperms [19,20]. However, the size and gene content varied even with the *Eragrostis* genus. Sequence alignment indicated that specific genes were lost in some cp genomes compared to *E. pilosa*. *E. tenellula*, for example, displayed loss of *trnfM*, *rps19, rps15*, *ndhH*, and *ycf68*. 

IR contraction and expansion causes length variation in angiosperm cp genomes [21]. Various gene migrations are common in most Poaceae species, such as the *ndhF* and *ndhH* genes, which are located at opposite ends of the SSC region and migrate toward the IR regions. The tribe Eragrostideae is classified in the PACMAD clade. Davis et al. (2010) [22] reported that in this clade, *ndhF* extended into the IR by < 30 nucleotides. Similar patterns of overlap were observed in this study, with the exception of *E. tenellula*, in which *ndhF* extended 53 nucleotides into the IRB region and *ndhH* was not present. The pattern in *En. oblongus* differed from other Eragrostideae species, with *ndhF* located entirely in the SSC. 

The phenomenon of codon usage bias refers to the unequal use of synonymous codons in an organism [23]. It is associated with the gene expression level and accuracy of translation [24], gene length [25], gene translation initiation signals, protein amino acid composition, protein structure, tRNA abundance, mutation frequency and patterns, and GC composition [26,27]. Consistent with previously reported cp genomes [28], the GC content of the Eragrostideae plastid genomes was reduced, suggesting an abundance of AT-rich intergenic regions in cp genomes. The AT-rich bias was strongest in the third codon position, also consistent with previously reported plastomes. 

As mentioned above, *E. pilosa* and *E. tef* are closely related species with similar morphology. We aligned their cp genome sequences to observe similarities and divergences at the genomic level. Between these two species, we observed variable regions that were mainly found in intergenic regions. We plotted the sequence identity using mVISTA using *E. pilosa* as a reference. The divergence was mostly located in the LSC region, with less divergence observed in the IR regions. However, divergence in this region was associated with the rrn cluster, containing *rrn16* and *rrn23*. Regions of high sequence divergence identified in this study could be used in deep phylogenetic analysis and as potential DNA barcodes. 

Taking the *E. walteri* to outside of the Chloridoideae, low genetic divergence was observed in *Eragrostis* compared to inter- generic divergence in Eragrostideae. Within the *Eragrostis* genus, comparatively low genetic divergence was observed between *E. tef* and *E. pilosa*, suggesting this inter-specific variation may account for *E. pilosa*'s many climatic adaptations. Evidence of chloroplast genes contribute to plant adaptation to the different environment has been discussed previously in *Najas flexilis* [29] and the Hawaiian genus *Schiedea* [30]. 

The proper taxonomic classification of *Eragrostis* species within Chloridoideae has been debated for many years, due to their similar morphologies, polyploid natures, and broad geographic distributions. However, this study does not suggest the need for a new infrageneric classification, but rather adds additional molecular information on the cp genomes for existing infrageneric classifications. Hilu and Alice (2001) [31] demonstrated that Eragrostideae species do not appear to be monophyletic, according to their *matK* sequences. However, Ingram and Doyle (2007) [3] used the plastid locus *rps16* and the nuclear gene *waxy* to demonstrate that *Eragrostis* species form a monophyletic group when a few segregate genera, including *Acamptoclados*, *Diandrochloa*, and *Neeragrostis* are included. In our data, *Eragrostis* species formed a monophyletic group, while *Enneapogon* and *Uniola* clustered separately. Meanwhile, Ingram et al. (2011) [7] demonstrated that *E. walteri* was misclassified in the Chloridoideae. Therefore, the placement of *E. walteri* in our phylogenetic tree is not surprising.

Alignment of the complete cp genomes demonstrated that *E. pilosa* and *E. minor* formed a clade and were sisters to *E. tef*. Additionally, it recovered a major *Eragrostis* clade and other genera. However, it is challenging to construct phylogenies for *Eragrostis*, because most known species are polyploid and the ploidy levels of many others remain unknown [3]. In BI trees based on highly divergent regions and 30 protein-coding genes, *E. pilosa*, *E. tef*, and *E. minor* clustered together. The similarities between *E. pilosa* and *E. tef* have been reported previously using morphological [32,33], cytological/biochemical [34,35] and molecular methods [10]. Based on cytological examinations, Tavassoli (1986) [35] suggested that *E. minor* and *E. pilosa* are close relatives of *E. tef*. Ingram and Doyle (2003) [10] showed that *E. minor* clustered with *E. pilosa* and *E.tef* with different progenitors. Therefore, extended sampling with several landraces of these close relatives will be necessary to place *E. pilosa* in its correct position. Our study highlights the utility of complete cp genome sequences, several protein-coding genes, and highly divergent regions for the phylogenetic analysis of Eragrostideae.

## 4. Materials and Methods

### 4.1. Plant Materials and DNA Sequencing

Fresh *E. pilosa* leaves were collected at Yan’an, Shaanxi, China (179° 36°39′20″ N 109° 24′26″ E). Total genomic DNA was extracted from silica-dried leaves using the modified cetyltrimethylammonium bromide (CTAB) method [36]. Purified DNA was used for paired-end library preparation with Illumina Paired-End DNA library Kit according to the manufacturer’s protocol (Illumina, CA, USA). Genomic DNA was sequenced on a HiSeq X Ten platform. Eight other species of Eragrostideae were used for comparative analysis (*E. tef* (NC_029413), *E. minor* (NC_029412), *E. setifolia* (NC_042832), *E. tenellula* (NC_042833), *E. walteri* (NC_035528), *En. caerulescens* (NC_042837), *En. oblongus* (NC_036682), *U. paniculata* (NC_036709)). 

### 4.2. Genome Assembly and Annotation 

We obtained high-quality reads from raw reads using the Trimmomatic tool [37]. The high-quality reads were then assembled using the GetOrganelle software (https://github.com/Kinggerm/GetOrganelle/blob/master/get_organelle_from_reads.py). Assembly was polished by mapping raw reads back to the retrieved sequence using the map reference tool in Geneious 10.0.5 (http://www.geneious.com). The generated consensus sequence was further adjusted by alignments with related species. Dual Organellar GenoMe Annotator online tool (https://dogma.ccbb.utexas.edu/) was used to annotate all protein-coding sequences and tRNA and mRNA genes [38]. Annotations of tRNA genes were confirmed with tRNAscan-SE [39]. Finally, the circular cp genome of *E. pilosa* was drawn with the online tool Organellar Genome DRAW (ORDRAW, https://chlorobox.mpimp-golm.mpg.de/OGDraw.html) [40]. The complete chloroplast genome of *E. pilosa* was deposited in GenBank with the accession number MN268502. 

### 4.3. Codon Usage Bias, Sequence Divergence, and K2P Genetic Distance Analyses

Codon usage bias and GC1, GC2, and GC3 contents were analyzed using 44 common protein-coding sequences. Each selected CDS met the following criteria: (1) a length > 300 bp; (2) presence of an ATG start codon and TAA, TGA, or TAG stop codon [41]. RSCU values and GC1, GC2, and GC3 contents were calculated using MEGA 6.0. [42]. 

Alignments of whole cp genomes were visualized with mVISTA [43]. Sequence divergence values were determined using the sliding window method in DnaSP v5.10. [44] with a 200-bp step size and an 800-bp window length. We estimated the K2P genetic distance for each protein-coding gene and the pairwise distance between each species using the K2P model in MEGA 6.0.

### 4.4. SSR Analysis

The MISA tool [45] was used to detect SSRs, with the parameters set to ten repeated units for mononucleotide SSRs, six repeated units for dinucleotide SSRs, and five repeated units for tri-, tetra-, penta-, and hexanucleotide SSRs. Shared repeats among *Eragrostis* species were determined by aligning the species to detect identical repeats located in homologous regions. The Primer3 online tool (http://primer3.ut.ee/) was used to design *E. pilosa* SSR primers for future studies [46]. IR border regions were visualized using the IRscope online program (https://irscope.shinyapps.io/irapp/).

### 4.5. Phylogenetic Analysis

All nine Eragrostideae species and *Eleusine indica* from Cynodonteae were used for phylogenetic analysis. Phylogenetic trees were derived from the following three data sets: (1) the complete cp genome sequences; (2) 30 protein-coding genes of > 600 bp length, to avoid sampling bias; and (3) a concatenation of highly divergent regions (*rps16-trnQ* + *trnL*+ *rbcL-psaI*). The analyses were based on the ML and BI methods. Coding sequences extracted from each sequence were aligned using MAFFT [47] and concatenated using Geneious software. The ML tree was constructed in MEGA 6.0 based on the Tamura-Nei model using a heuristic search for initial trees. Bootstrap analysis was performed with 1,000 replicates. A discrete gamma distribution was used with four categories (+G, parameter = 0.3797). Initial ML tree(s) were obtained using the neighbor joining and BIONJ algorithms. BI analysis was conducted using MrBayes 3.2 [48] with a General Time Reversible substitution model. Two independent Markov chain Monte Carlo chains were run for 1.5 million generations, with a subsampling frequency of 200 generations.

## 5. Conclusions

In this study, we sequenced the complete cp genome sequence of *E. pilosa*, a grass belonging to tribe Eragrostideae. Specific genome features and SSRs were identified and compared with those of other Eragrostideae species. This study is the first complete cp genome comparison of Eragrostideae species, using all complete Eragrostideae cp genomes available in the NCBI database to date. Phylogenetic analysis demonstrates that cp genome data sets can be used to resolve Eragrostideae phylogeny through broad sampling. This study makes a significant contribution to the literature because it provides new information on the genetic relationships between *Eragrostis* species that could be used for further species identification and phylogenetic analysis.

## Figures and Tables

**Table 1:** Chloroplast genome features of *E. pilosa* and other Eragrostideae species.

Species	Acc. No	Size	LSC	SSC	IR	Number of Genes	Number Of Protein Coding Genes	Number of tRNA Genes	Number of rRNA Genes
*Eragrostis pilosa*	MN268502	134,815	80,100	12,661	21,027	132	85	39	8
*Eragrostis tef*	NC_029413	134,435	79,802	12,581	21,026	132	85	39	8
*Eragrostis minor*	NC_029412	135,023	80,316	12,577	21,065	133	85	40	8
*Eragrostis setifolia*	NC_042832	134,928	80,416	12,494	21,009	127	81	38	8
*Eragrostis tenellula*	NC_042833	130,773	79,387	12,598	19,394	124	78	38	8
*Eragrostis walteri*	NC_035528	137,056	81,763	12,697	21,298	129	83	38	8
*Enneapogon caerulescens*	NC_042837	133,231	78,883	12,410	20,969	127	81	38	8
*Enneapogon oblongus*	NC_036682	133,433	78,839	12,546	21,024	133	85	40	8
*Uniola paniculata*	NC_036709	135,322	80,643	12,595	21,042	131	83	40	8

**Table 2:** GC content of the Eragrostideae cp genome sequences. GC content (g) whole cp genome (c) 44 analyzed CDS (1) (2) (3) first, second, and third codon positions, respectively.

Species	Analyzed CDS	GC (g)	GC(c)	GC1	GC2	GC3
*E. pilosa*	44	38.3	39	48.0	39.0	29.9
*E. tef*	44	38.3	39	48.0	39.1	29.9
*E. minor*	44	38.2	39	48.0	39.1	30.0
*E. setifolia*	44	38.3	39.1	48.1	39.1	30.3
*E. tenellula*	44	38.4	39	47.9	39.1	30.0
*E. walteri*	44	38.3	39.1	48.1	39.0	30.3
*En. caerulescens*	44	38.3	39	48.0	39.1	30.0
*En.oblongus*	44	38.3	39	48.0	39.1	29.8
*U. paniculata*	44	38.3	39.1	48.0	39.1	30.0

**Table 3:** SSR distribution among Eragrostideae cp genomes. CSSR; compound SSR.

Species	Total SSR	CSSR	A/T	C/G	AT/TA	LSC	SSC	IRa	IRb
*E. pilosa*	45	6	42	3	-	40	1	2	2
*E. tef*	50	7	47	2	1	47	1	1	1
*E. setifolia*	40	3	38	2	-	33	3	2	2
*E. tenellula*	49	2	45	3	1	42	3	2	2
*E. walteri*	52	7	51	-	1	47	3	1	1
*E. minor*	41	5	38	2	1	36	1	2	2
*En. caerulescens*	44	2	41	-	3	39	1	2	2
*En. oblongus*	45	2	42	-	3	40	1	2	2
*U. paniculata*	50	8	49	-	1	50	-	-	-

## Supplementary Materials

Supplementary tables. https://www.mdpi.com/2223-7747/8/11/485/s1.
